# Multiscale Solutions to Quantitative Systems Biology Models

**DOI:** 10.3389/fmolb.2019.00119

**Published:** 2019-10-30

**Authors:** Nehemiah T. Zewde

**Affiliations:** Department of Bioengineering, University of California, Riverside, Riverside, CA, United States

**Keywords:** mathematical models, systems biology, complement system, molecular dynamics, Brownian dynamics, ordinary differential equations, innate immunity

## Introduction

Systems biology implements a variety of statistical, computational and mathematical techniques to understand how networks of biological systems work together to achieve a function (Westerhoff and Palsson, 2004; Wolkenhauer, 2014). Systems biology is a multi-scale field, as it has no fixed scale in the context of a biological response or cascade, where an ensemble of proteins, cofactors and small molecules concertedly act to achieve function. This is the case of fundamental pathophysiological networks, such as epidemiological responses with host and pathogens (Hillmer, 2015). Understanding the network of interactions that mediate these systems is of the utmost importance for deciphering the mechanisms associated with multifactorial diseases, as well as to address fundamental biological questions. This knowledge can be used for translational research and application in biomedicine (McGillivray et al., 2018). The multi-scale nature of systems biology calls for a multifaced description to bridge the system scale at the cellular level to the molecular scale of individual macromolecules.

Among the important biological cascades responsible for severe diseases, we focus here on the complement system, which is an effector arm of the immune system that eliminates pathogens, helps in maintaining host homeostasis, and forms a bridge between innate and adaptive immunity (Bennett et al., 2017; Reis et al., 2019). Complement is composed of three pathways known as alternative, classical and lectin that work in concert to achieve its function (Schatz-Jakobsen et al., 2016a). The complex network of proteins and other macromolecular entities composing the complement system represents an ideal case to build a systems biology workflow predicting the system's response in immunity against invading pathogens, and how under complement deficiencies this same system mediates different pathologies. Here, we report on the development of systems biology predictive models, which describe the intricate biochemical networks and the crosstalk among other elements of the immune system. We also show how the integration of multiscale modeling techniques can help for improving the predictive model, while also providing mechanistic information at the molecular level.

Complement dysfunction is associated with several diseases. Among others, the complement components have been associated with neurodegenerative disorders including Alzheimer and Parkinson diseases; as well as multiple sclerosis (Mastellos et al., 2019). Moreover, mutations of complement proteins have been linked to the etiology of renal diseases (De Vriese et al., 2015; Ricklin et al., 2016), while individuals with complement deficiencies develop severe infections, such as meningitis, bacteremia and pneumonia caused by microorganisms, such as *Streptococcus pneumoniae, Neisseria meningitidis*, and *Staphylococcus aureus* (Skattum et al., 2011). Clearly, while a proper activation of the complement system is associated with a wide spectrum of beneficial effects, dysfunctional states are associated with severe consequences. Considering that the function of the complement system is regulated by a network of multiple components, whose concerted activity underlies a variety of diseases, accurate models of the interaction network would greatly help therapeutic strategies (Ricklin et al., 2018).

## Mathematical Models of the Complement System

**Figure 1:**
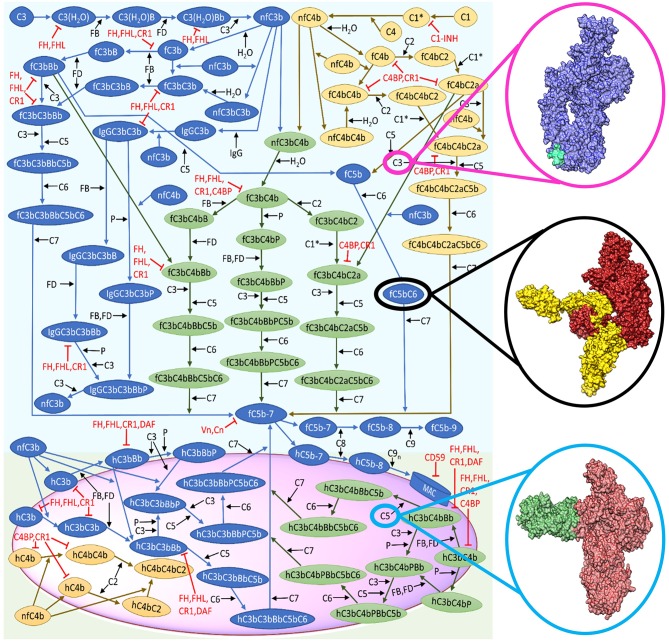
Reduced biochemical network of the complement system (alternative and classical). The representative surface of host or pathogen is shown in magenta. Complement activation start in the fluid phase, whereas the crosstalk between the alternative and classical pathways is shown in green. The cascade of reactions will propagate to the surface and terminate by the formation of the membrane attack complex (MAC). This figure is adapted from Zewde and Morikis (2018). Structural representation of C3 (blue) with compstatin (cyan) shown in magenta circle (Janssen et al., 2005, 2007). Black circle denotes the surface representation of C5b in firebrick coloring and C6 in yellow (Hadders et al., 2012). Surface representation of C5 (red) and eculizumab (H- and L-chain in green) shown in light blue circle (Schatz-Jakobsen et al., 2016b).

The complexity of the complement system arises from the mechanistic function of numerous proteins and related biochemical reactions within the complement pathways (Figure 1). For instance, complement is composed of more than 60 proteins that circulate in plasma and bound to cellular membranes of host cells that work to mediate different phases (fluid and solid) of immunity (Liszewski et al., 2017). This multi-phasic interaction between complement proteins forms the basis of the intricate biochemical networks and numerous crosstalk with different compartments of the immune system, such as pentraxins (C-reactive protein, serum-amyloid P, and long pentraxin 3) and the coagulation cascade (Amara et al., 2008; Ma and Garred, 2018).

In this complex scenario, mathematical models using ordinary differential equation (ODE) emerged as a powerful tool to elucidate the dynamics of the complement system. Indeed, ODEs can be used to generate predictive models of complex biological processes involving metabolic pathways, protein-proteins interactions, and tumor growth (Ilea et al., 2012; Dubitzky et al., 2013; Rohrs et al., 2018). In defining a biological network in a quantitative manner, ODE models can enable to predict concentrations, kinetics and behavior of the network components, building hypotheses on disease causation, progression and interference, which can be tested experimentally (Enderling and Chaplain, 2014). In line with this, models of the complement system based on ODEs have been designed to mechanistically deconstruct segments of the complement system under homeostasis and infection (Hirayama et al., 1996; Korotaevskiy et al., 2009; Liu et al., 2011; Zewde et al., 2016; Sagar et al., 2017; Lang et al., 2019).

To further these efforts, we recently generated an expanded ODE model that predicts the complement biomarker levels under the states of homeostasis, disease, and drug intervention (Zewde and Morikis, 2018). By using the reaction network in Figure 1, we generated a system of ODEs to describe the bi-phasic nature of the complement system: (i) initiation (fluid phase); (ii) amplification and termination (pathogen surface); and (iii) regulation (host cell and fluid phase). The ODE representation is shown below:

$$\begin{array}{l} \frac{{dC}_{i}}{dt}=\;\overset{x_{i}}{\underset{y=1}{\sum }}\sigma_{ij}f_{j} \end{array}$$

where variable *C*_*i*_ represents the concentration of an individual complement protein/complex, *x*_*i*_ denotes the number of biochemical reactions associated with complement *C*_*i*_ for the *y*^*th*^ reaction. Moreover, σ_ij_, denotes stoichiometric coefficients and *f*_*j*_ is a function that describes how the concentration *C*_*i*_ changes with the biochemical reactions of the reactants/products and parameters, within the given timeframe.

Building on this basic concept, we have designed a model of the complement system that incorporates pathological conditions by reducing the regulatory kinetic rates constants and lowering blood plasma concentrations (Zewde and Morikis, 2018). By applying this model, it is possible to perform *in silico* mutation by perturbing a complement protein and its binding partner and examine how it translates into the global dynamics of the complement pathway activation and regulation. As a consequence, this enables to generate patient specific models provided clinical data, predicting the effect of a specific mutation within the entire system. For instance, disorders, such as C3 glomerulonephritis and dense-deposit disease are associated with a mutation that affects the complement regulatory protein factor H (FH) (Nester and Smith, 2016). This mutation results in low plasma levels of FH and subsequently leads to host cell damage due to under-regulation of the alternative pathway. By measuring patient's FH level, this value can be used to reparametrize the starting concentration of FH in the ODEs model and, subsequently, examine how the mutation affects activation and regulation of the alternative pathway (Zewde and Morikis, 2018). The ODE mathematical models can also be used to identify novel therapeutic targets, which can be object of experimental validations to assess their capability to interfere with the complement system. In this respect, one strategy, called “global sensitivity,” enables to identify which set of kinetic parameters is important in the network of the complement system. In parallel, the “local sensitivity” analysis can help in pinpointing critical complement components that mediate the output of activation or regulation (examples in Liu et al., 2011; Zewde et al., 2016; Sagar et al., 2017). ODE models are also useful if kinetic data is available for known inhibitors. Indeed, ODEs can be used to perform comparison studies on how different therapeutic targets perform under disease-based perturbations. In our previous work (Zewde and Morikis, 2018), we incorporated two complement inhibitors known as compstatin, C3 inhibitor (Figure 1, magenta circle), and eculizumab, C5 inhibitor (Figure 1, light blue circle), and examined how they regulated a disease state mediated by FH. Our model showed both inhibitors performed differently in regulating an over-active complement system (disease state). Compstatin was shown to potently regulate early-stage complement biomarkers, whereas eculizumab over-regulates late-stage biomarkers. From these results, our model indicated the need for patient-tailored therapies depending on how disease associated mutations manifest in the complement cascade. Altogether, ODE models can be utilized to mechanistically translate convoluted biological reaction-networks, reparametrized for patient specific modeling, and identify novel therapeutic targets under pathological conditions.

## Multiscale Solutions to the Challenges of ODE Models

Building on ODE models that predict how the molecular interactions mediate immunity and disease, our group has expanded the ODEs approach to model the pathways of the complement system as a whole. In this respect, one of the main challenges is represented by the lack of kinetic parameters, thereby significantly hindering our modeling efforts. For instance, we are currently building a comprehensive complement model that includes all three pathways (Figure 1), immunoglobulins (IgG and IgM) and pentraxins. This system, which comprises 670 differential equations with 328 kinetic parameters, is used to examine the interplay between complement activation and an immune evasive bacteria *Neisseria meningitidis*. However, 140 of our kinetic parameters are unknown and estimation of these parameters is challenging, due the limited availability of experimental data.

To overcome these challenges, multi-scale approaches can aid in alleviating some of these burdens by performing simulations to predict association rate constants. For example, Brownian dynamics (BD), milestoning and molecular dynamics (MD) can be used to predict the kinetic and conformational requirements of binding (Ermak and McCammon, 1978; Huber and McCammon, 2010; Votapka and Amaro, 2015). MD enables to follow the motions of macromolecules over time by integrating Newton equation of motion. As opposite, BD simulates a system based on an overdamped Langevin equation of motion, enabling the study of diffusion dynamics and obtaining association rates for a given process (Ermak and McCammon, 1978). Novel hybrid schemes, such as SEEKR combines multiscale approaches of MD, BD, and milestoning to estimate kinetic parameters of association and dissociation rate constants (Votapka et al., 2017).

We have already initiated this bridge between systems biology and multi-scale approaches by performing molecular dynamics and electrostatics studies on the complement complex C5bC6 (Figure 1, black circle) (Zewde et al., 2018). Our analysis identified three binding sites and critical salt bridges formed between C5b and C6. Building on this first study, Brownian dynamics simulations will aid into the prediction of kinetic parameters associated with C5bC6 complex formation, which will subsequently be inserted into our ODE model. As a further useful approach, in the cases where complete structural data are absent, homology models using computational tools, such as MODELLER (Webb and Sali, 2016) or SWISS-MODEL (Waterhouse et al., 2018) can be used as a supplement. This step can be followed by the utilization of protein docking tools like HADDOCK (Dominguez et al., 2003) or ClusPro (Kozakov et al., 2017) to generate potential complement complexes. Finally, top ranked structures can then be a subject of the multi-scale approaches mentioned above to estimate unknown kinetic parameters.

## Summary and Perspectives

Here, we described the current efforts to model the complexity of systems biology, by building predictive models based on ODEs. The multi-scale nature of this field, as characterized by a network of proteins, cofactors and small molecules concertedly acting to achieve function, calls for a multiscale description bridging the macromolecular level to the systems level. Here, we described our investigations aimed at modeling the complex biological response of the complement system, which plays a prominent role in host defense, homeostasis, and disease. We showed how ODEs models can provide description of the network of interactions at the system level, while multiscale simulations methods can complement this approach providing a description at the macromolecular level.

ODE models of the complement system have elucidated key mechanisms of immune system function and regulation. These mathematical models show promise for the investigation of patient specific diseases and for the identification of therapeutic interventions under pathological conditions. Despite these advantages, modeling efforts are continuously challenged by the lack of kinetic parameters needed to generate and simulate ODEs models. A multi-scale approach—harnessing methods, such as Brownian and molecular dynamics—is promising to address some of these challenges by predicting unknown kinetic parameters to be utilized in quantitative models of the complement system. In addition to multi-scale estimations, high performance computing has made it possible to simulate large biological structures (Casalino et al., 2018; Palermo et al., 2018). This opens scientific avenues in the frontier of modeling entire biochemical networks, including the complement system, such merging the molecular level perspective to the system (i.e., cellular) scale.

## Author Contributions

NZ designed the study and wrote the manuscript.

### Conflict of Interest

The author declares that the research was conducted in the absence of any commercial or financial relationships that could be construed as a potential conflict of interest.
